# Effect of ivabradine-induced heart rate reduction on flow-mediated dilation measured with high-sensitivity ultrasound in patients with stable coronary heart disease

**DOI:** 10.1186/1476-7120-12-5

**Published:** 2014-01-31

**Authors:** Nicoline Jochmann, Franziska Schröter, Fabian Knebel, Robert Hättasch, Christine Gericke, Karl Stangl, Gert Baumann, Verena Stangl

**Affiliations:** 1 Asklepios Klinik St. Georg, Klinische und interventionelle Angiologie, Lohmühlenstraße 5, 20099 Hamburg, Germany; 2 Medizinische Klinik m.S. Kardiologie und Angiologie, Charité – Universitätsmedizin Berlin, Charité Campus Mitte, Charitéplatz 1, 10117 Berlin, Germany; 3 Institut für Biometrie und Klinische Epidemiologie, Charité – Universitätsmedizin Berlin, Charité Campus Benjamin Franklin, Hindenburgdamm 30, 12203 Berlin, Germany

**Keywords:** Flow-mediated dilation, Heart rate, Endothelial function, Ivabradine

## Abstract

**Background:**

Experimental data suggests that exclusive heart rate reduction with ivabradine is associated with the amelioration of the endothelial function. Since it is presently unknown whether this also applies to humans, the aim of this pilot study was to investigate whether heart rate reduction with ivabradine modulates the endothelial function in humans with an established coronary heart disease.

**Methods:**

Using high-sensitivity ultrasound, we analysed the flow-mediated (FMD) and nitro-mediated dilation (NMD) of the brachial artery in 25 patients (62.9 ± 8.4 years) with a stable coronary heart disease and a resting heart rate of ≥70 beats per minute (bpm). To assess acute effects, measurements were performed before and 4 hours after the first intake of ivabradine 7.5 mg. Sustained effects of an ivabradine therapy (5 mg to 7.5 mg twice daily) were investigated after 4 weeks.

**Results:**

We found a significant decrease in heart rate, both 4 hours after the intake of 7.5 mg of ivabradine (median -8 [interquartile range (IQR) -14 to -4] bpm) and after 4 weeks of twice daily intake (median -10 [IQR-17 to -5] bpm) (p < 0.05). However, the FMD did not change significantly: neither after first dose of ivabradine nor after sustained therapy (baseline FMD: median 5.0 [IQR 2.4 to 7.9]%; FMD 4 hours after 7.5 mg of ivabradine: median 4.9 [IQR 2.7 to 9.8]%; FMD after 4 weeks of ivabradine therapy: median 6.1 [IQR 4.3 to 8.2]%). No significant changes of the NMD were observed. In regression analysis, the heart rate and FMD did not correlated, irrespective of the ivabradine intake (r^2^ = 0.086).

**Conclusion:**

In conclusion, in our study heart rate reduction through ivabradine does not improve the endothelial function in patients with a stable coronary heart disease. Moreover, we found no correlation between the heart rate and the endothelial function.

## Background

Numerous epidemiologic studies suggest that an elevated heart rate (HR) is associated with an increase in cardiovascular morbidity and mortality [1-5]. This negative prognostic value of a higher HR appears to be independent of traditional cardiovascular risk factors [1,6,7]. In addition, experimental and clinical evidence indicates that an increased HR contributes to the pathogenesis of atherosclerosis. Oxidative stress, expanded levels of low shear-stress periods and increased mechanical burden are discussed as underlying mechanisms [8-10]. There are some clues that the HR modulates the endothelial function as well; however, results are controversial. Both positive as well as negative correlations of the HR with the FMD as parameter of endothelial-dependent vasodilation have been described [11-14].

Ivabradine (Iva), a selective inhibitor of I_f_ channels, reduces the resting and exercise HR without affecting cardiac contractility, blood pressure, intracardiac conduction, or ventricular repolarisation [15-17]. It therefore offers the unique opportunity to assess effects of an exclusive HR reduction [5]. Since it is presently unknown whether changes in the HR have an impact on the endothelial function in humans, the aim of our study was to investigate effects of a HR reduction in response to Iva on the endothelial function measured by the FMD of the brachial artery.

## Methods

### Study population

Patients with a stable coronary heart disease and betablocker intolerance (chronic obstructive lung disease, asthma, psoriasis, hypotension, Raynaud’s syndrome) or with an insufficient HR reduction during betablocker treatment were included in the study from January 2010 to September 2011. The main inclusion criterion was a resting HR of ≥70 beats/min in two consecutive ECGs. Exclusion criteria were a recent (<2 months) myocardial infarction or revascularisation procedures (<4 weeks), ventricular or atrioventricular pacing, atrial fibrillation or flutter. We required routine laboratory parameters to lay within the normal range. It was also necessary that blood lipids, blood pressure, body mass index (BMI) and HbA1c as well as glucose levels demonstrated good adjustment according to guidelines for secondary prevention in coronary heart disease [18]. The study was approved by the Charité University Hospital Ethics Committee (CEC), and participants provided their written informed consent. The study was carried out in accordance with the Code of Ethics of the World Medical Association (Declaration of Helsinki).

### Study design

**Figure 1:**
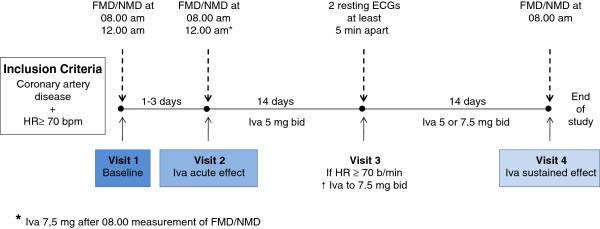
**Flow-chart of the study protocol.** HR: heart rate; Iva: ivabradine; FMD: flow-mediated dilation; NMD: nitro-mediated dilation.

This prospective, non-randomized study was designed to evaluate the acute and sustained effects of Iva on the endothelial function. At the first study visit (control), baseline FMD/NMD data were assessed in the morning after a 12-hour overnight fast and 4 hours thereafter. At the second study visit, FMD/NMD measurements were performed before and 4 hours after a first dose of Iva (7.5 mg) to investigate acute effects on the endothelial function. After the morning measurement, patients consumed a standardized meal, which consisted of two wheat rolls, one package of low fat cream cheese (1,5% fat per 100 g) and half a liter of still water. Patients thereafter received Iva (5 mg) twice daily for four weeks. In case of a HR of ≥60/min at day 14 (documented in two resting ECGs at least 5 min apart), the Iva dosage was increased to 7.5 mg twice daily. After 4 weeks of Iva intake, FMD/NMD values were assessed again at 08.00 a.m. (sustained effect). Blood pressure and HR were documented simultaneously at each time point (Figure 1).

### Flow-mediated dilation (FMD) and nitro-mediated dilation (NMD)

The endothelial function was measured by high-resolution vascular ultrasound with a 13-MHz linear-array transducer (Sonoline Antares, Siemens, Germany), as recently described [19]. The endothelium-dependent FMD was assessed by measuring the change in the brachial artery diameter during reactive hyperaemia after cuff occlusion of the forearm for 5 min, according to established guidelines [20,21]. To improve the accuracy and reproducibility of the measurements, an attached device fixed the ultrasound probe at one site of the brachial artery during the entire period of measurements. Changes in the arterial diameter were measured every 15 sec up to 3 min. The FMD was defined as the maximum percentage change in diameter compared with the baseline measurement. The nature of the stimulus and conditions of the measurements of FMD were chosen to obtain a nitric oxide-dependent response after a brief period of shear stress in the brachial artery [21]. The endothelium-independent vasodilation (NMD) was determined after sublingual application of nitroglycerin spray (0.4 mg). The increase in diameter of the brachial artery was measured from min 1 to 6, and the NMD was defined as the maximum percentage change in diameter compared with the baseline measurement. Ultrasound images were digitized online and saved. Analyses of diameter changes were conducted offline (Tom Tec Imaging Systems, Germany) by an investigator blinded to subject treatment. Intra- and interobserver variations for FMD max (%) were 0.97 and 0.91 and for NMD max (%) 0.99 and 0.98 in 20 by random chosen studies.

### Statistical analysis

Values for the FMD and HRs in tables, text and figures are presented as median values (Q1 – Q3). The baseline characteristics are means ± standard deviation. All statistical tests were two-sided with the level of significance at 5%. The non-parametric test design was chosen because of the small sample size. The paired design was considered by using the Wilcoxon test. Comparison between the FMD and HR took place with regression analysis. Statistical analyses were performed by using IBM SPSS Statistics Version 20.

## Results

**Figure 2:**
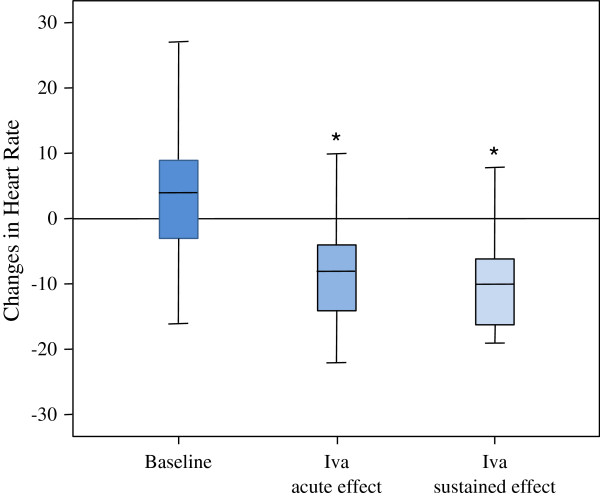
**Heart rate during study protocol.** Values are presented as median (Q1-Q3).^.^Iva: ivabradine; baseline: difference between heart rate at 08.00 and 12.00 at study visit 1 (without Iva); Iva acute effect: difference between heart rate before and 4 hours after Iva; Iva sustained effect: difference between heart rate before first dose of Iva (study visit 2) and after 4 weeks of Iva intake twice daily (10 to 15 mg). *p < 0.05 for Iva acute effects vs. baseline and Iva sustained effects vs. baseline.

**Figure 3:**
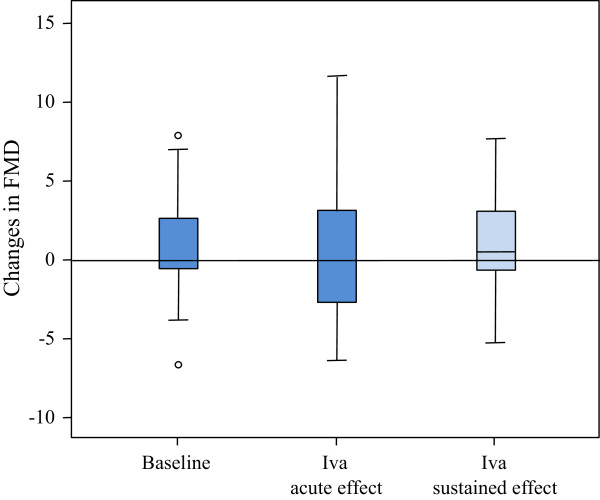
**FMD during study protocol.** Values are presented as median (Q1-Q3). Iva: ivabradine; FMD: flow-mediated dilation; baseline: difference between FMD at 08.00 and 12.00 at study visit 1 (without Iva); Iva acute effect: difference between FMD before and 4 hours after Iva; Iva sustained effect: difference between FMD before first dose of Iva (study visit 2) and after 4 weeks of Iva intake twice daily (10 to 15 mg).

**Figure 4:**
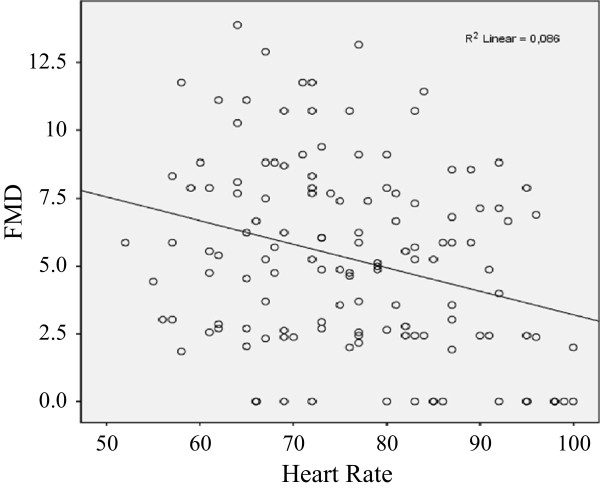
**Heart rate and FMD.** Regression analyses of heart rate and FMD irrespective of ivabradine intake. FMD: flow-mediated dilation.

A total of 25 patients were included in the study arm to investigate the acute effects of Iva on the endothelial function. In 17 of these patients, the sustained effects of an up to four weeks long Iva therapy were assessed. Patients’ baseline characteristics and demographic data are depicted in Tables 1 and 2. The mean age was 62.9 ± 8.4 years and the mean BMI 27.7 ± 4.9 kg/m^2^. 44% of patients were receiving betablocker treatment. Medians of FMD and NMD measurements over the whole study period are shown in Table 3. We found a significant decrease in the HR both 4 hours after the intake of 7.5 mg of Iva (-8 [-14 to -4] bpm) and after 4 weeks of twice daily intake (-10 [-17 to -5] bpm), shown in Figure 2. However, the FMD did not change significantly: neither after the first dose of Iva nor after sustained therapy (baseline FMD: 5.0 [2.4 to 7.9]%; FMD 4 hours after 7.5 mg of Iva: 4.9 [2.7 to 9.8]%; FMD after 4 weeks of Iva therapy: 6.1 [4.3 to 8.2]%) (Figure 3). Iva failed to modulate the endothelial function regardless of the presence or absence of concomitant betablocker therapy (data not shown). Moreover, the degree of HR reduction had no influence on Iva effects (data not shown). No significant changes of NMD were observed. Median values for FMD and NMD measurements are depicted in Table 3. In regression analysis, HR and FMD did not correlate, irrespective of Iva intake (r^2^ = 0.086) (Figure 4).

**Table 1:** Baseline characteristics

	**Mean ± SD**
Age (years)	62.9 ± 8.4
BMI (kg/m^2^)	27.7 ± 4.9
Heart rate (bpm)	82.4 ± 10.8
RR syst. (mmHg)	126.7 ± 16.2
RR diast. (mmHg)	76.8 ± 9.0
LVEF (%)	54.8 ± 8.6
Glucose (mg/dl)	109.5 ± 34.3
Cholesterol (mg/dl)	177.8 ± 50.7
LDL (mg/dl)	106.0 ± 40.1
HDL (mg/dl)	56.4 ± 25.9
Triglycerides (mg/dl)	132.2 ± 54.9

BMI = body mass index; RR syst. = systolic blood pressure; RR diast. = diastolic blood pressure; LVEF = left ventricular ejection fraction; LDL = low-density lipoprotein; HDL = high-density lipoprotein.

**Table 2:** Demographic characteristics

	**n (%)**
Male	19 (76)
Smokers	6 (24)
Hypertension	22 (88)
Diabetes	10 (40)
Dyslipidaemia	20 (80)
Myocardial infarction	10 (40)
CABG/PCI	20 (80)
ICD	2 (8)
Angina pectoris	17 (68)
β-blocker	11 (44)

CABG = coronary artery bypass graft; PCI = percutaneous coronary intervention; ICD = implantable cardiac defibrillator.

**Table 3:** Median values of FMD and NMD

	**Baseline**		**Acute effect**		**Sustained effect**
	**8.00 a.m.**	**12.00 a.m.**	**8.00 a.m.**	**12.00 a.m.**	**8.00 a.m.**
FMD_base_ (mm)	3.8 [3.3-4.2]	3.8 [3.4-4.2]	3.7 [3.3-4.2]	3.6 [3.3-4.1]	3.6 [3.4-4.3]
FMD_max_(mm)	3.9 [3.5-4.3]	4.0 [3.6-4.3]	3.8 [3.5-4.3]	3.9 [3.5-4.3]	3.8 [3.6-4.4]
FMD_max_ (%)	4.8 [2.4-6.5]	5.3 [2.2-7.5]	5.0 [2.4-7.9]	4.9 [2.7-9.8]	6.1 [4.3-8.2]
NMD_base_ (mm)	3.6 [3.2-4.2]	4.1 [3.4-4.2]	3.8 [3.1-4.4]	4.1 [3.3-4.4]	3.7 [3.3-4.2]
NMD_max_ (mm)	4.4 [4.1-4.8]	4.4 [4.2-4.9]	4.4 [3.9-5.0]	4.4 [4.1-5.1]	4.4 [4.1-4.9]
NMD_max_ (%)	19.1 [13.2-25.7]	17.0 [12.5-23.7]	16.7 [13.0-24.6]	17.3 [9.8-23.7]	21.1 [13.5-29.6]

Values are presented as median values (Q1 – Q3). FMD_base_: diameter of brachial artery before hyperaemic stimulus; FMD_max_ : maximum dilation of brachial artery after hyperaemic stimulus, shown in mm as maximum percentage change in brachial artery diameter compared with FMD_base_; NMD_base_: diameter of brachial artery before application of 0.4 mg nitroglycerine; NMD_max_: maximum dilation of brachial artery after application of 0.4 mg nitroglycerine, shown in mm and as maximum percentage change in brachial artery diameter compared with FMD_base._. Baseline: FMD at 08.00 and 12.00 at study visit 1 (without ivabradine); Acute effect: FMD before and 4 hours after first intake of 7,5 mg ivabradine; Sustained effect: FMD after 4 weeks of ivabradine intake twice daily (10 to 15 mg).

## Discussion

The main finding of our study is that Iva treatment is not associated with a significant amelioration of the endothelial function in patients with stable coronary artery disease. Although the HR declined by 10%, no changes in the endothelial-dependent vasodilation assessed non-invasively by high-sensitivity ultrasound of the brachial artery were observed: neither acutely nor after one month of treatment with Iva. Even in the patient subgroup with the highest HR at baseline and with the greatest reduction in response to Iva, we found no significant impact on the endothelial function.

These results are in line with two recently published trial in humans which also did not find an improvement of the FMD after Iva treatment of diabetic patients and of patients with angina pectoris despite a profound reduction of the HR [22,23]. These two studies in humans stand, however, in contrast to experimental data obtained on several occasions in mice with lipid-induced endothelial dysfunction, which reveals that endothelial-dependent vasodilation improves in response to a HR reduction with this I_f_-channel blocker [8-10,24]. Drouin reported in 2008 that Iva prevents deterioration of the endothelial dilator function of renal and cerebral arteries associated with dyslipidemia in mice expressing human ApoB-100 [9]. Similarly, in apolipoprotein E-deficient mice fed a Western-type diet, Iva improved the endothelial function in aortic rings, exerted potent antioxidative effects and reduced atherosclerotic plaque formation [8]. These effects were independent of blood pressure or lipid lowering [8]. Very recently, Schirmer et al. showed that Iva enhanced arteriogenesis and increased eNOS expression, NO availability and endothelial-dependent relaxation in a murine hindlimb model of endothelial dysfunction [24]. HR reduction has been incriminated as the main underlying mode of action, since Iva had no direct effect on the endothelium: neither in cultured endothelial cells nor in isolated rings from ApoE-/- mice [24].

The reasons for the inconsistent results between our data in humans and in animal models are unclear; however, species differences may be of importance. The HR is usually below 100 beats/min in humans and above 600 beats/min in small animals such as mice. Harzheim et al. reported that HCN4 channels serve to regulate the HR in adult mice only during and after stress and that, accordingly, effects of Iva may not be directly compared to data obtained in humans [25,26].

Principally, the paradigm must be questioned that there is a comprehensive correlation between the level of the HR and the endothelial function. As mentioned before, human studies assessing this correlation have yielded diverging results [11,13,14,26]. In a community-based cohort of 2883 participants, a positive correlation between the FMD and HR was found, which suggests that an increased HR is associated with an improved endothelial function [13]. In contrast, these two parameters correlated inversely in smaller studies. In these investigations, a bias of the chosen triggers to modulate the HR (e.g., physical activity or mental stress) cannot be ruled out [11,14]. One study in pacemaker patients found no effects of different cardiac pacing protocols (80–120 bpm) on FMD [12]. To obtain further insights, we analysed in our patient group whether the HR correlates with FMD. The fact that this was not the case (Figure 4) may explain why an HR reduction in response to Iva treatment failed to modulate the endothelial function.

### Limitations

Several limitations of our study must be considered. First, because of the relatively small sample size, the study may be not sufficiently powered to assess significant differences. Second, the absence of a placebo-controlled group may have biased some results.

Third, the brachial diameter was not measured continuously as we didn’t use a semiautomatic system for analyses of FMD and NMD. Nevertheless intra- and interobserver variations for FMD and NMD showed a good accuracy of our methodology.

## Conclusion

In conclusion, our data did not support the concept that a reduction of the HR is beneficial with regard to the amelioration of the endothelial function. There is no acute or sustained effect on the flow-mediated dilation measured by high-frequency ultrasound despite a significant HR reduction.

## Abbreviations

BMI: Body mass index; bpm: Beats per minute; CEC: Charité University Hospital Ethics Committee; FMD: Flow-mediated dilation; HR: Heart rate; Iva: Ivabradine; NMD: Nitro-mediated dilation; IQR: Interquartile range.

## Competing interests

The Medizinische Klinik mit Schwerpunkt Kardiologie und Angiologie, Charité – Universitätsmedizin Berlin, Campus Mitte has received a grant from Servier (France). The funders had no role in the study design, data collection and analysis, decision to publish, or preparation of the manuscript.

## Authors’ contributions

NJ, VS and FS have designed and coordinated the study. FS, NJ, FK, RH and CG have analysed the data. NJ and FS have written the manuscript. FS and RH have performed the measurement of the endothelial function for the study. NJ, FS, FK, VS, KS and GB performed the planning of the study and the patient selection. All authors have read and approved the final manuscript.
